# Chromosomal Distribution of Genes Conferring Tolerance to Abiotic Stresses Versus That of Genes Controlling Resistance to Biotic Stresses in Plants

**DOI:** 10.3390/ijms21051820

**Published:** 2020-03-06

**Authors:** Richard R.-C. Wang

**Affiliations:** USDA-ARS Forage and Range Research Lab, Utah State University, Logan, UT 84322-6300, USA; richard.wang@usda.gov

## 1. Introduction

Tolerance to abiotic stresses caused by environmental conditions can prevent yield loss in crops for sustaining agricultural productivity [1]. Resistance to biotic stresses caused by diseases and insects can prevent or reduce yield loss in crops [2]. For each crop or plant species, there are many abiotic threats, such as changes in temperature, soil salinity/alkalinity, water shortage, and soil contaminants, as well as biotic challenges from pathogens (bacteria, viruses, and fungi), insects, and nematodes. Plants need to possess genes conferring tolerance to these abiotic stresses to adapt to the changing environment, due to global climate changes, in which they are growing. Due to the coevolution of plants and stress-causing organisms [3], plants need to possess multiple resistance genes to deal with the rise of new virulence in stress-causing organisms. Plant breeders are constantly looking for new resistance genes to combat evolving organisms that pose a threat to susceptible crops. As a result, plant geneticists have identified many resistance genes in various crops, and molecular geneticists have developed molecular markers for most of those genes. Similarly, researchers are investigating plant mechanisms and underlying genetic systems involved in plant tolerance to abiotic stresses, hoping to breed crops resilient to adverse environmental conditions.

With the advent of whole-genome sequencing in many important crops, it is time to map the detailed chromosomal locations of known genes that are involved in tolerance to various abiotic stresses as well as in the resistance to biotic stresses in important plant species. In the Special Issue, "Mapping Abiotic Stress-Tolerance Genes in Plants" of International Journal of Molecular Sciences, 21 papers, including two reviews and 19 research articles, were published [4,5,6,7,8,9,10,11,12,13,14,15,16,17,18,19,20,21,22,23,24]. Eleven research articles [3,25,26,27,28,29,30,31,32,33,34] were published in the Special Issue “Mapping Plant Genes that Confer Resistance to Biotic Stress.”

In this editorial, I firstly express my appreciation to all authors for their contribution to the two Special Issues. Secondly, I will compare the chromosomal distribution patterns of genes for the two types of stresses that plants faced (Table 1 and Table 2). The evidence obtained supports my long-held hypothesis that genes conferring resistance to biotic stresses are more likely to be located in the distal portion of chromosomes than the proximal portion in order to adapt to the host-pest coevolution. On the other hand, abiotic-stress tolerance genes should have a lower ratio of distal to proximal distribution than that for biotic stresses to maintain the stability of genes regulating plant growth and development. Knowing the relationship between gene functions and their chromosomal distribution patterns, plant breeders can select the most appropriate and efficient method to improve crops for withstanding stresses and ensuring productivity and food security.

## 2. Chromosomal Distribution Patterns of Genes for Abiotic-Stress Tolerance vs. Biotic-Stress Resistance

Studying abiotic-stress tolerance, the authors of these 21 articles in this Special Issue covered *Hordeum vulgare*, *Gossypium hirsutum*, *Pyrus pyrifolia*, *Oryza sativa*, *Glycine max*, *Fragaria vesca*, *Cucumis sativus*, *Dianthus caryophyllus*, *Brassica oleracea*, *B. napus*, *Sorghum bicolor*, *Triticum aestivum*, *Zea mays*, *Raphanus sativus*, and the model plant *Arabidopsis thaliana* (Table 1). The abiotic stresses studied include cold, heat, drought, salt, iron deficiency, nitrogen deficiency, UV irradiation, DNA damage, reducing agent, phytohormones (GA, SA, JA, ABA, ethylene, 2,4-D, and NAA), and heavy metals (cadmium, nickel and cobalt). Two [6,7] of the 21 articles did not present information on the chromosomal locations of genes for abiotic-stress tolerance, and one [18] did not map the BocMBF1c gene to the target species *B. oleracea* but did locate the orthologous gene identified in *A. thaliana* to the proximal section of chromosome 3. 

Many transcription factor gene families (TFs) were studied in the majority of these 21 articles [6,8,9,11,13,14,15,16,17,18,19,20,23,24]. Various putative stress-related and hormone-responsive cis-acting regulatory elements were identified in the promotor of these TFs. “The cis-regulatory sequences are linear nucleotide fragments of non-coding DNA with the main role of regulating gene expression and in turn, controls the development and physiology of an organism” [35]. Therefore, variations among members of TFs observed in those studies might account for the varying regulation of gene expression in different organs and tissues or at different developmental stages to respond to different stresses.

Among the 11 articles in the Special Issue on plant genes conferring resistance to biotic stresses [3,25,26,27,28,29,30,31,32,33,34], seven articles reported results from single resistant genes (or QTL) for crops and plant species, including soybean, rice, wheat, *Dasypyrum villosum*, *Aegilops searsii*, *Capsicum annuum*, and *Vitis quinquangularis*. The other four articles [27,29,30,32] analyzed multiple QTLs or genomic regions for one or more diseases. 

For genes controlling tolerance to abiotic stresses, an averaged 2.2 to 1 ratio of distal to proximal chromosomal distribution was obtained from the 21 articles (Table 1). In comparison, the 11 articles on genes conferring resistance to biotic stresses resulted in a 3.3 to 1 ratio (Table 2). Therefore, 77% of genes conferring resistance to biotic stresses were located in the distal section of chromosomes, while 69% of those for abiotic-stress tolerance were distally located. This slightly higher number of genes in the distal section of chromosomes is advantageous for plant adaptation, because genetic variability generated from the high recombination rate in distal recombination hotspots enables plants to deal with environmental changes and new virulent pests. 

## Figures and Tables

**Table 1:** Chromosomal distribution of genes controlling tolerance to abiotic stresses.

					Chromosome Arm			
Plant Species	Genes	Stress	Mechanisms	Chromosome	distal	proximal	total	Reference
Barley *(Hordeum vulgare*)	P-Type ATPase (*HvPAA1*) gene in a single QTL *qShCd7H*	Cadmium	Plasma membrane-localized cation-transporting ATPase.	7H	0	1	1	Wang et al. 2019 [4]
Cotton (*Gossypium hirsutum*)	ROS-network genes (*CSD1, APX1, APX2, MDAR1, GPX4-6-7, FER2, RBOH6, RBOH11, FRO5, AOX, GLR, and PER, etc.*)	Cold, heat, dehydration, salt	ROS network-mediated signal pathway.	Nine each of A and D genomes	21	15	36	Xu et al. 2019 [5]
Pear (*Pyrus pyrifolia*)	C-repeat binding factor (*PpyCBF1 to 6*) genes	Low temperature, salt, drought, and abscisic acid (ABA).	ABA-dependent and -independent pathways, ROS and antioxidant.	1, 4, 6, 7, 14 and one scoffold.	-	-	-	Ahmad et al. 2019 [6]
Rice (*Oryza sativa*)	AHA2, FRO2, IRT1, FIT, FRD3, FPN1, YSL2, VIT1, NRAMP3/4	Iron deficiency.	Iron acquisition from soil, iron transport fromroots to shoots, and iron storage in cells.	-	-	-	-	Zhang et al. 2019 [7]
*Arabidopsis thaliana*	Stress-Responsive NAC Transcription Factor (*LlNAC2*) of tiger lily	Cold, drought, salt stresses, and abscisic acid (ABA).	DREB1/ZFHD4/CBF-COR interaction and ABA signaling pathways.	1S (in *Arabidopsis*)	1	0	1	Yong et al. 2019a [8]
*Arabidopsis thaliana*	MYB related homolog (*LlMYB3*) of tiger lily	Cold, drought, and salt stresses, ABA treatment.	LlCHS2 and anthocyanin biosynthesis pathway.	5L (in *Arabidopsis*)	1	0	1	Yong et al. 2019b [9]
*Soybean (Glycine max*)	four QTLs for resistance to high-intensity UV-B irradiation (UVBR12-1, 6-1, 10-1, and 14-1)	UV-B irradiation (high light, heat, dehydration),	Possibly, actin-binding spectrin like protein interacting with membrane phosphoinositides in cellular signaling for defense.	12, 6, 10, and 14	2	2	4	Yoon et al. 2019 [10]
Woodland Strawberry (*Fragaria vesca*)	GIBBERELLIN-INSENSITIVE (GAI), REPRESSOR OF GA1-3 (RGA) and SCARECROW (SCR) protein (*FveGRAS*) genes	Cold, heat, and GA3 treatments.	Stolon formation, fruit ripening and abiotic stresses.	All 7	25	10	35	Chen et al. 2019 [11]
*Arabidopsis thaliana*	N-MYC Downregulated Like Proteins (NDL1, NDL2, NDL3) interacting with ANN1, SLT1, OAS-TL, ARS27A, RGS1, AGB1	Heat, cold, dehydration, DNA damage, reducing agent, increased intracellular calcium, metal ions like cadmium, nickel and cobalt, hormones.	N-MYC Downregulated Like Proteins (NDLs) interacting with G-Proteins in signal transduction in response to drought, heat, salinity and light intensity.	All 5	5	4	9	Katiyar and Mudgil 2019 [12]
Soybean (*Glycine max*)	calmodulin binding transcription activator gene (*GmCAMTA*)	Drought.	Calmodulin binding Ca-CaM-CAMTA-mediated stress regulatory mechanisms.	8 out of 20 (5, 7, 8, 9, 11, 15, 17, 18)	10	5	15	Noman et al. 2019 [13]
Cotton (*Gossypium hirsutum*)	nodule inception-like protein (*Gh*NLP) genes	Nitrogen deficiency	Promoters of NLP genes interact with stress-associated transcription factors and be targeted by many miRNAs.	All 26	91	14	105	Magwanga et al. 2019 [14]
Cucumber (*Cucumis sativus* L.)	GAGA-binding BASIC PENTACYSTEINE (BPC) transcription factor genes (*CsBPCs*)	Salt, drought, cold, heat, ABA, SA, JA, ETH, 2,4-D, GA.	Germination, growth and development, as well as responses to abiotic stresses and plant hormones.	3 of 7 (2, 5, 7)	3	1	4	Li et al. 2019 [15]
Carnation (*Dianthus caryophyllus*)	Heat shock transcription factors (Hsfs)	Heat, drought, cold, salt, ABA, SA.	Promoters included various cis-acting elements that were related to stress, hormones, as well as development processes, controlling reactive oxygen species homeostasis, and ABA-mediated stress signaling.	17 scaffolds	10	7	17	Li et al. 2019 [16]
Cotton (*Gossypium hirsutum*)	Histone Acetyltransferase (HAT) Gene family	Salt, drought, cold, heavy metal, DNA damage, ABA, NAA.	Affect cotton growth, fiber development, and stress adaptation by regulation of chromatin structure, activate the gene transcription implicated in various cellular processes.	8 of 26 (A-5,6,8,11 and D-5,6,10,11)	16	2	18	Imran et al. 2019 [17]
Chinese kale (*Brassica oleracea*)	multi-protein bridging factor (MBF) 1c (*BocMBF1c*)	Heat stress: cellular response to hypoxia, ethylene-activated signaling pathway, positive regulation of transcription, DNA-templated response to abscisic acid heat, and water deprivation.	BocMBF1c contains three heat shock elements (HSEs) and helix-turn-helix (HTH) domains, regulating ABRFs, SA, trehalose, and ET thermal resistance-related pathways by binding with CTAGA, including *DREB2A.*	not presented; ortholog on chromosome 3 of Arabidopsis thaliana^*^	-; 0	-; 1 *	-; 1 *	Zou et al. 2019 [18]
Soybean (*Glycine max*)	Pentatricopeptide-repeat (PPR) proteins DYW subgroup genes; *GmPPR4*	Drought and salt.	Delayed leaf rolling; higher content of proline (Pro); and lower contents of H2O2, O2, and malondialdehyde (MDA); increased transcripts of several drought-inducible genes.	all 20 chromosomes; *Gm*PPR4 is on chromosome 1 distal end	143	36	179	Su et al. 2019 [19]
Bread wheat (*Triticum aestivum*)	WRKY transcription factor superfamily genes; TaWRKY13^*^	Salt, drought, ABA, cold.	More root development, increased proline (Pro) and decreased malondialdehyde (MDA) contents.	all chromosomes except 4B and 7B; 2A	33	24; 1^*^	57	Zhou et al. 2019 [20]
oilseed rape (*Brassica napus*)	Fructose-1,6-bisphosphate aldolase (FBA) gene family (*BnaFBA*)	Salt, heat, drought, *Sclerotinia sclerotiorum* infection, and strigolactones (SLs) treatments.	Processes of glycolysis, gluconeogenesis, and Calvin cycle; Various cis-acting regulatory elements existed within the promoter regions of BnaFBA genes.	19 on 15 *B. napus* chromosomes; 3 others to 2 random chromosomes (two on the An chromosomes and one on the Cn chromosome)	7	15	22	Zhao et al. 2019 [21]
Sorghum (*Sorghum bicolor*)	stay-green QTL	Drought and heat.	N/C supply-demand, photosynthesis, water use efficiency, leaf anatomy, mineral and sugar transportation, senescence.	All 7	10	7	17	Kamal et al. 2019 [22]
Wheat (*Triticum aestivum*)				1A, 2A, 4A, 5A, 1B, 2B, 3B, 4B, 4D, 7D	10	8	18	
Rice (*Oryza sativa*)				2 to 12	9	18	27	
Maize (*Zea mays*)				1, 2, 3, 5, 6, 8, 9	12	11	23	
Barley (*Hordeum vulgare*)				All 7	4	6	10	
Soybean (*Glycine max*)	Calcium-dependent protein kinases (CDPKs) genes; *GmCDPK3* ^*^	Drought and salt.	Increased proline (Pro) and chlorophyll contents and decreased malondialdehyde (MDA) content.	12 of 20 (1 to 6, 10, 11, 14, 16, 18, 19)	14; 1 ^*^	3	17	Wang et al. 2019 [23]
Radish (*Raphanus sativus*)	Lipoxygenases (LOXs) gene family *RsLOX*	Abiotic (drought, salinity, heat, and cold) and biotic (*Plasmodiophora brassicae* infection) stress conditions.	three tandem-clustered RsLOX genes are involved in responses to various environmental stresses via the jasmonic acid pathway.	5 of 9 (2, 5, 7, 8, 9)	5	6	11	Wang et al. [24]
Total					432	196	628	
Ratio					2.2:1			

* The chromosome position is not in Brassica but is in Arabidopsis.

**Table 2:** Chromosomal distribution of genes controlling resistance to biotic stresses.

					Chromosome Arm			
Plant Species	Genes	Biotic Stress	Mechanisms	Chromosome	distal	proximal	total	Reference
*Glycine max*	*RpsX*	Phytophthora root rot (PRR) caused by *Phytophthora sojae* (Rps).	A 144-bp insertion in the Glyma.03g027200 sequence resulted in two additional leucine-rich (LRR) encoding fragments.	3	1	0	1	Zhong et al. 2019 [25]
*Oryza sativa*	QTL *qFfR9* with 35.15% additive effect	Bakanae disease (BD), caused by the fungal pathogen *Fusarium fujikuroi*.	Eight genes in the QTL may be candidate genes for BD resistance.	9	1	0	1	Kang et al. 2019 [26]
*Triticum aestivum*	18 QTL	Karnal bunt caused by *Neovossia indica.*	QTL are associated with NBS-LRR proteins, Serine/threonine-protein kinase, Protein Kinase family protein, Kinase family protein, Receptor-like kinase, C2H2-like zinc finger protein, F-box domain containing protein, Glycosyltransferase and Transcription factor gene families.	1D, 2B, 2D, 4A, 4B, 5A, 5B, 6A, 6B, 7B, 7D	15	3	18	Gupta et al. 2019 [27]
*Oryza sativa*	Lesion mimic mutant (LMM) gene *LMM24*	*lmm24* exhibited enhanced resistance to rice blast fungus *Magnaporthe oryzae* and up-regulation of defense response genes.	Receptor-like cytoplasmic kinase 109 (OsRLCK109) leads to dark brown lesions in leaves and growth retardation due to enhanced ROS accumulation.	LOC_Os03g24930 on chromosome 3	0	1	1	Zhang et al. 2019 [28]
*Triticum aestivum*	3 QTL for stripe rust resistance	Stripe rust, caused by *Puccinia striiformis* f. sp. *tritici.*	QTL on 1B may be *Yr29 (an APR gene); the minor* QTL on 2Al may be a new stripe rust resistance locus; Qyr.saas-7B could be in the same locus of QYr.nsw-7B from Tiritea.	1BL, 2AL, 7BL	3	0	3	Yang et al. 2019 [29]
*Triticum aestivum*	124 genomic regions associated with various diseases; several genes in those significant genomic regions had gene annotations suggesting their involvement in disease resistance.	wheat rusts (leaf; *Puccinia triticina*, stem; *P. graminis* f.sp. *tritici*, and stripe; *P. striiformis* f.sp. *tritici*) and crown rot (*Fusarium* spp.); cereal cyst nematode (*Heterodera* spp.); and Hessian fly (*Mayetiola destructor*).	Five genes were annotated as the leucine-rich repeat protein family and six genes were annotated as the F-box family protein, which were also reported to be involved in abiotic stress tolerance such as drought; Calcium-binding protein; ARM repeat superfamily protein; Elongation factor 1 alpha; Peroxidase; WAT1-related protein/EamA-like transporter family.	21 chromosomes	97	27	124	Bhatta et al. 2019 [30]
*Dasypyrum villosum* to *Triticum aestivum*	*Sr52*	Wheat stem rust caused by *Puccinia graminis* f. sp. *Tritici.*	Resistant to stem rust Ug99 races.	6V#3L bin FL 0.92–1.00 to 6AL.	1	0	1	Li et al. 2019 [31]
*Triticum aestivum*	Seven significant additive QTLs for TS resistance explaining 2.98 to 23.32% of the phenotypic variation; five QTLs explaining 5.24 to 20.87% of SNB resistance	Tan Spot (induced by *Pyrenophora tritici-repentis*) and Septoria Nodorum Blotch (caused by *Parastagonospora nodorum*).	Quantitative resistance: fungus *P. tritici-repentis* isolates produce at least three host-selective toxins (HSTs), Ptr ToxA, Ptr ToxB and Ptr ToxC that interact with products of specific host sensitivity genes located on chromosome arm 5BL, 2BS., and 1AS, respectively, to cause disease.	TS (1A, 1B, 5B, 7B and 7D); SNB (1A, 5A, and 5B)	7	5	12	Singh et al. 2019 [32]
*Capsicum annuum*	A major QTL *qRRs-10.1*	bacterial wilt (BW), caused by *Ralstonia solanacearum.*	A cluster of five predicted R genes and three defense-related genes.	chromosome 10	0	1	1	Du et al. 2019 [33]
*Aegilops searsii* to *Triticum aestivum*	*Pm57*	Powdery mildew caused by *Blumeria graminis* f. sp. *tritici*.	Ten genes that are putative R genes which includes six coiled-coil nucleotide-binding site-leucine-rich repeat (CNL), three nucleotide-binding site-leucine-rich repeat (NL) and a leucine-rich receptor-like repeat (RLP) encoding proteins.	2S^s^#1, fraction length 0.72–0.87	1	0	1	Dong et al. 2020 [34]
*Vitis quinquangularis*	Transcription Factor *VqMYB14*	bacterial flagellin peptide flg22 and harpins (glycine-rich and heat-stable proteins that are secreted through type III secretion system in gram-negative plant-pathogenic bacteria).	The promoter of *VqMYB14 is* induced by the elicitors flg22 to confer basal immunity (also called pathogen-associated molecular pattern (PAMP)-triggered immunity, PTI) and triggered by harpin to confer effector-triggered immunity (ETI). Overexpression of *VqMYB14* enhance the main stilbene contents and expression of stilbene biosynthesis genes.	chromosome 7	0	1	1	Luo et al. 2020 [3]
Total					126	38	164	
Ratio					3.3:1			
